# Analytical performance of species-targeted quantitative PCR for intra-abdominal candidiasis in critically ill patients: a proof-of-concept post hoc analysis from the pBDG2 multicenter study

**DOI:** 10.1186/s13054-026-05882-5

**Published:** 2026-02-10

**Authors:** Emmanuel Novy, Arnaud Schaeffer, Maxime Nguyen, Frédéric Dalle, Julien Pottecher, Valérie Letscher-Bru, Guillaume Louis, Charlotte Stephan, Amandine Luc, Marie Machouart, Anne Debourgogne

**Affiliations:** 1 Université de Lorraine, CHRU-Nancy, Anesthésie-réanimation et médecine périopératoire, Nancy, 54000 France; 2 SIMPA, Université de Lorraine, Vandoeuvre-Lès-Nancy, 54500 France; 3 Université Bourgogne Europe, CHU Dijon Bourgogne, INSERM U.M.R 1231, Department of anesthesiology and intensive care medicine, Center for Translational and molecular Medicine, Lipness Team, Dijon, 21000 France; 4 U.M.R. PAM Univ Bourgogne Europe, Institut Agro Dijon, INRAE, UFR Sciences de Santé, Équipe Aliment, Fermentation, Interaction, Microbiote, AFIM, 21070 Dijon, France; 5 Laboratoire de Parasitologie-Mycologie, CHU Dijon, 21070 Dijon, France; 6 Service d’Anesthésie-Réanimation & Médecine Péri-Opératoire, Hôpital de Hautepierre, Hôpitaux universitaires de Strasbourg, Strasbourg, 67200 France; 7 Faculté de médecine, Maïeutique et Science de la Santé, UR3072, FMTS, FHU DATA-SURGE, Université de Strasbourg, Strasbourg, 67000 France; 8 Laboratoire de Parasitologie et Mycologie Médicale, Plateau Technique de Microbiologie, Hôpitaux Universitaires de Strasbourg, Strasbourg, 67000 France; 9 Service de réanimation polyvalente, CHR Metz-Thionville, Metz, 57000 France; 10 Laboratoire de microbiologie, CHR Metz-Thionville, Metz, 57000 France; 11 Unité de méthodologie, data management et statistiques, DRCI, CHRU de Nancy, Nancy, 54000 France; 12 Université de Lorraine, CHRU-Nancy, Microbiologie, Nancy, F-54000 France

**Keywords:** Intra-abdominal candidiasis, Critically ill patient, Diagnosis, PCR candida

## Abstract

**Background:**

Intra-abdominal candidiasis (IAC) is a severe form of invasive candidiasis that affects critically ill patients and is associated with a high mortality rate and persistent diagnostic delays. Conventional fungal culture from peritoneal fluid remains slow and has insufficient sensitivity. Molecular assays promote early diagnosis, but data from critically ill populations remain scarce.

**Objectives:**

We evaluated the diagnostic performance of the OLM CandID Real-Time PCR assay directly applied to peritoneal fluid samples obtained from a previously established multicenter cohort of critically ill patients with suspected or confirmed IAC.

**Methods:**

OLM CandID Real-Time PCR is a quantitative PCR assay for detecting *C. albicans*,* C. glabrata*,* C. parapsilosis*,* C. tropicalis*,* C. krusei*, and *C. dubliniensis*. Binary PCR results were compared with those of fungal culture as the reference standard. Secondary analyses analyzed quantitative PCR values to better understand culture-negative/PCR-positive discordances using ROC-derived cycle threshold (Ct) cutoffs.

**Results:**

Fifty-seven patients, mainly with postoperative peritonitis, were included. Among the culture-positive samples, *C. albicans* was predominant. Using binary interpretation, the PCR achieved 100% sensitivity (95% CI 100–100) and 60% specificity (95% CI 38–81); the positive and negative predictive values were 82% (95% CI 71–93) and 100% (95% CI 100–100), respectively. In the secondary Ct-based analysis, ROC-derived threshold determination was feasible for *C. albicans* (*n* = 35) and *C. tropicalis* (*n* = 12). For *C. albicans* and *C. tropicalis*, the ROC AUC values were 0.92 (95% CI 0.75-1) and 0.90 (95% CI 0.66-1), respectively, and applying the optimal Ct cutoffs (30.75 and 34.63 respectively) yielded 100% specificity and 100% positive predictive value for both species.

**Conclusions:**

OLM CandID Real-Time PCR applied directly to peritoneal fluid demonstrated strong analytical performance for the detection of *Candida* in critically ill patients with suspected IAC. The assay showed high concordance with culture results, supporting its feasibility for rapid microbiological assessment. Discordant PCR-positive/culture-negative results were mainly associated with high Ct values, likely reflecting low fungal burden, detection of non-viable DNA, or analytical over-detection. These findings are exploratory and highlight the need for prospective studies to determine the clinical relevance of quantitative PCR results and their role in guiding antifungal management.

**Trial registration:**

The study was registered with ClinicalTrials.gov (ID number 07005258, first registered on March 6, 2025).

**Supplementary Information:**

The online version contains supplementary material available at 10.1186/s13054-026-05882-5.

## Introduction

Intra-abdominal candidiasis (IAC) represents one of the most severe manifestations of invasive candidiasis in the intensive care unit (ICU) [1–3]. Despite advances in source control and antifungal therapy, it remains associated with substantial morbidity and mortality [4, 5]. Recent expert reviews report that IAC accounts for 10–30% of intra-abdominal infections in the ICU and is linked to high mortality, particularly in patients with postoperative peritonitis or gastrointestinal leaks [6–8]. More broadly, invasive candidiasis in critically ill patients is associated with significant mortality, reflecting persistent delays in diagnosis despite the use of modern antifungal agents.

The diagnosis of IAC remains challenging [9–12]. The reference standard is fungal culture from peritoneal fluid or intra-abdominal specimens. However, culture-based methods are slow, often requiring several days, and suffer from limited sensitivity, especially after antifungal exposure [13–15]. Blood cultures are positive in less than 10% of IAC episodes, underscoring the limitations of systemic sampling in this compartmentalized infection.

Given these shortcomings, expert groups increasingly emphasize the need for accelerated, nonculture diagnostic strategies [16, 17]. Molecular assays, such as CandID Real-time PCR (OLM diagnostics; Braintree, UK), have already demonstrated strong diagnostic performance for candidemia [18–20], providing rapid and sensitive detection of *Candida* species. However, the utility of PCR assays in IAC remains largely unexplored, and data on peritoneal fluid specimens are scarce [21–24]. Moreover, the implementation of these techniques is hampered by limited standardization, heterogeneous performance across platforms, cost considerations, and a lack of clinical validation in IAC [14].

A frequently cited concern with molecular diagnostics is the potential for overdetection and false-positive results, particularly when fungal burdens are low. Importantly, *Candida* is not a biologically uniform genus: different species display distinct ecological behaviors, virulence, growth kinetics, and invasive potential, resulting in heterogeneous fungal burdens and clinical relevance in invasive infections. Recent expert discussions on fungal taxonomy and diagnostics highlight that species-level differentiation reflects true biological and clinical heterogeneity rather than purely nomenclatural changes [25]. Consequently, a given PCR cycle threshold may not carry the same diagnostic implication across species. The quantitative nature of real-time PCR offers an opportunity to address this limitation by distinguishing clinically meaningful infection from low-level, nonpathogenic signals. In this context, assessing whether species-specific cycle threshold (Ct) values can refine diagnostic interpretation represents a critical research question.

Building on this rationale, the present study aimed to evaluate the diagnostic performance of OLM CandID Real-Time PCR applied directly to peritoneal fluid in critically ill patients with suspected or confirmed IAC and to explore whether species-specific Ct values could help interpret discordant culture-negative/positive PCR results.

## Materials and methods

### Study design: setting

This study is a secondary, post hoc analysis based on a biological collection and clinical dataset generated during a previously conducted French multicenter prospective cohort [7]. The present secondary analysis was not pre-specified at the time of the parent study. It was registered with ClinicalTrials.gov (ID number 07005258) on March 6, 2025, in accordance with current recommendations for transparency in secondary analyses.

### Participants

The parent cohort, previously published [7, 26], prospectively enrolled 199 critically ill adult patients admitted to the ICU with suspected or confirmed intra-abdominal candidiasis (IAC) between January 2020 and December 31, 2022, during which the biological sample collection used in the present analysis was constituted. Inclusion required (i) clinical suspicion of intra-abdominal infection; (ii) peritoneal fluid obtained by intraoperative sampling, percutaneous aspiration, or drainage from a recently (<24 h) inserted abdominal drain; and (iii) suspicion or confirmation of *Candida* involvement according to contemporary IAC definitions. Within the parent cohort, 87 patients (44%) had confirmed IAC, all classified as *proven* cases according to the FUNDICU consensus [11]. No additional clinical exclusion criteria were applied beyond those of the parent study. Only one peritoneal fluid sample per patient was included in the analysis.

### Sample selection for this study

Following completion of the parent study, peritoneal fluid samples were initially stored at −80 °C in the institutional biological resource center. For the present secondary analysis, samples were subsequently retrieved and stored at −20 °C during the PCR analytical period. Freeze–thaw cycles were minimized, and each sample was thawed only once prior to PCR analysis. For this pilot study, only 60 PCR tests were available. As the evaluated OLM CandID Real-Time PCR had never been applied to peritoneal fluid, a subset of samples was first used in an initial optimization phase prior to the final diagnostic evaluation. This phase included both culture-positive and culture-negative samples since fungal culture has imperfect sensitivity. Because some specimens had been partially consumed during the parent study, only samples with a remaining volume of ≥ 600 µL were eligible. As many of the remaining culture-positive samples as possible were retained for the analysis, provided they met the volume requirement. For control purposes, culture-negative samples were randomly selected to represent 50% of the number of retained culture-positive samples, which also met the same volume and availability criteria. This approach was chosen to maximize inclusion of culture-positive samples while remaining within the limit of 60 PCR tests for the study, and to maintain a manageable and representative control set.

### Microbiological and clinical data

For each sample, the following data were available: peritoneal bacterial and fungal cultures, peritoneal 1.3-beta-d-glucan concentration (using the beta-glucan test^®^ from Wako Fugifilm, Neuss, Germany), serum 1.3-beta-d-glucan concentration (using the beta-glucan test or the Fungitell^®^ beta-D-glucan assay from Associated of Cape Cod, East Falmouth, Inc., United States of America) and OLM CandID Real-Time PCR results. While peritoneal 1.3-beta-d-glucan (pBDG) measurement was systematically planned in the parent study, serum 1.3-beta-d-glucan (sBDG) testing reflected routine ICU practice and was therefore not available for all patients, resulting in missing sBDG data for some samples. Fungal culture was performed using standard mycological methods and specifically aimed at the detection and identification of *Candida* species. For each sample, data on demographics, comorbidities, type of intra-abdominal infection, organ support, and mortality were also presented.

### PCR assay

The CandID Real-Time PCR (OLM Diagnostics, Braintree, UK) is a quantitative real-time PCR assay that can detect six clinically relevant *Candida* species, namely, *C. albicans*,* C. glabrata*,* C. parapsilosis*,* C. tropicalis*,* C. krusei*, and *C. dubliniensis*, including an internal extraction control [19]. In accordance with current fungal taxonomy, *C. glabrata*,* C. krusei* and *C. kefyr* have been reclassified as *Nakaseomyces glabratus*,* Pichia kudriavzevii* and *Kluyveromyces marxianus*, respectively. However, to ensure clarity for a critical care readership, the historical *Candida* nomenclature is retained throughout the manuscript, with updated taxonomic names provided at first mention in accordance with recent expert recommendations [25]. The assay provides species-level detection and quantitative measurements expressed as cycle threshold (Ct) values. For this study, the assay was adapted to the ELITech InGenius instrument (ELITechGroup SAS, Puteaux, France), a fully automated sample-to-result molecular diagnostics platform. DNA extraction was performed using magnetic-capture technology with a 600 µL input volume, following the manufacturer’s recommendations. Each sample included an internal extraction control to validate extraction efficiency and ensure the absence of PCR inhibitors. Negative and positive controls were included in every run.

Ct values were automatically determined by the instrument, and the manufacturer does not provide a predefined Ct cutoff for qualitative interpretation. Therefore, laboratory personnel were blinded to both culture and clinical results during analysis. This setup allows for an unbiased assessment of Ct distributions and enables exploration of potential diagnostic thresholds in the present study. The assays were performed at the Mycology Department of Nancy University Hospital (France) between July and August 2025.

## Objectives

### Primary objective

To evaluate the diagnostic performance of the OLM CandID Real-Time PCR assay in peritoneal fluid by comparing binary PCR results (positive/negative) with fungal culture results used as the reference standard.

### Secondary objectives

To explore the potential contribution of quantitative OLM CandID Real-Time PCR in interpreting discordant culture-negative/PCR-positive cases, we considered that such discordances may reflect higher analytical sensitivity of PCR, detection of non-viable fungal DNA, contamination, or low fungal burden below the limit of culture detection. In this context, the term low-level fungal DNA is used to describe the detection of *Candida* DNA in peritoneal fluid in the absence of culture growth, without implying physiological colonization of the peritoneal cavity. Species-specific cycle threshold (Ct) values were examined to provide additional insight, and exploratory Ct cutoffs were derived where sample size permitted.

To explore the potential contribution of peritoneal 1.3-beta-d-glucan concentration in interpreting discordant culture-negative/PCR-positive cases.

### Primary analysis (binary diagnostic performance)

The primary analysis assessed the diagnostic performance of the CandID Real-Time PCR using a qualitative binary approach, consistent with its intended clinical use.

PCR results were classified as positive or negative based solely on the presence or absence of an amplification signal, without applying any Ct threshold, as no manufacturer-recommended Ct cutoff is provided for qualitative interpretation.

PCR results were compared with fungal culture results, which served as the reference standard and were categorized as positive or negative. Sensitivity, specificity, positive predictive value (PPV), and negative predictive value (NPV) were calculated with 95% confidence intervals. Agreement between PCR and culture was assessed using Cohen’s kappa coefficient derived from a 2 × 2 contingency table.

### Secondary analysis (quantitative PCR and Ct threshold exploration)

A secondary, exploratory analysis was conducted to investigate whether quantitative PCR data (Ct values) could provide additional insight into discordant culture-negative/PCR-positive results.

Ct value distributions were examined and compared between concordant samples (culture-positive/PCR-positive) and discordant samples (culture-negative/PCR-positive). Where sample size permitted, receiver operating characteristic (ROC) curve analyses were performed to explore the ability of Ct values to discriminate culture-positive cases from discordant cases.

Exploratory Ct thresholds were derived using the Youden index for species or aggregated groups with sufficient events. These thresholds were applied in sensitivity analyses to assess how reclassification of high-Ct PCR-positive results might affect diagnostic performance metrics.

This quantitative analysis was not intended to redefine the qualitative interpretation of the assay, but rather to generate hypotheses regarding the interpretation of PCR-positive/culture-negative results in the absence of an established Ct cutoff.

Peritoneal 1.3-beta-d-glucan (pBDG) concentration was evaluated to assist in the interpretation of discordant PCR-positive/culture-negative samples. The concentrations of pBDG were dichotomized using a previously established cutoff (≤45 pg/mL vs. >45 pg/mL) [7] and compared with fungal culture as the reference standard. The sensitivity, specificity, PPV, and NPV with 95% confidence intervals were calculated. No formal comparative analysis between serum and peritoneal BDG, or between BDG and PCR as index tests, was performed, as the primary objective of this study was to evaluate the PCR assay.

### Statistical analysis

All eligible stored samples were included in this exploratory post hoc study. Descriptive statistics are presented as counts and percentages for categorical variables and as the means ± standard deviations or medians with interquartile ranges for continuous variables, as appropriate. Statistical tests such as the chi-square or Fisher’s exact test were used to compare categorical variables, while Student’s t test or the Wilcoxon test was applied to continuous variables. All tests were two-sided with a significance level of 0.05. Analyses were performed by an independent statistician using SAS version 9.4 (SAS Institute, Inc., Cary, NC).

## Results

### Participants

**Fig. 1:**
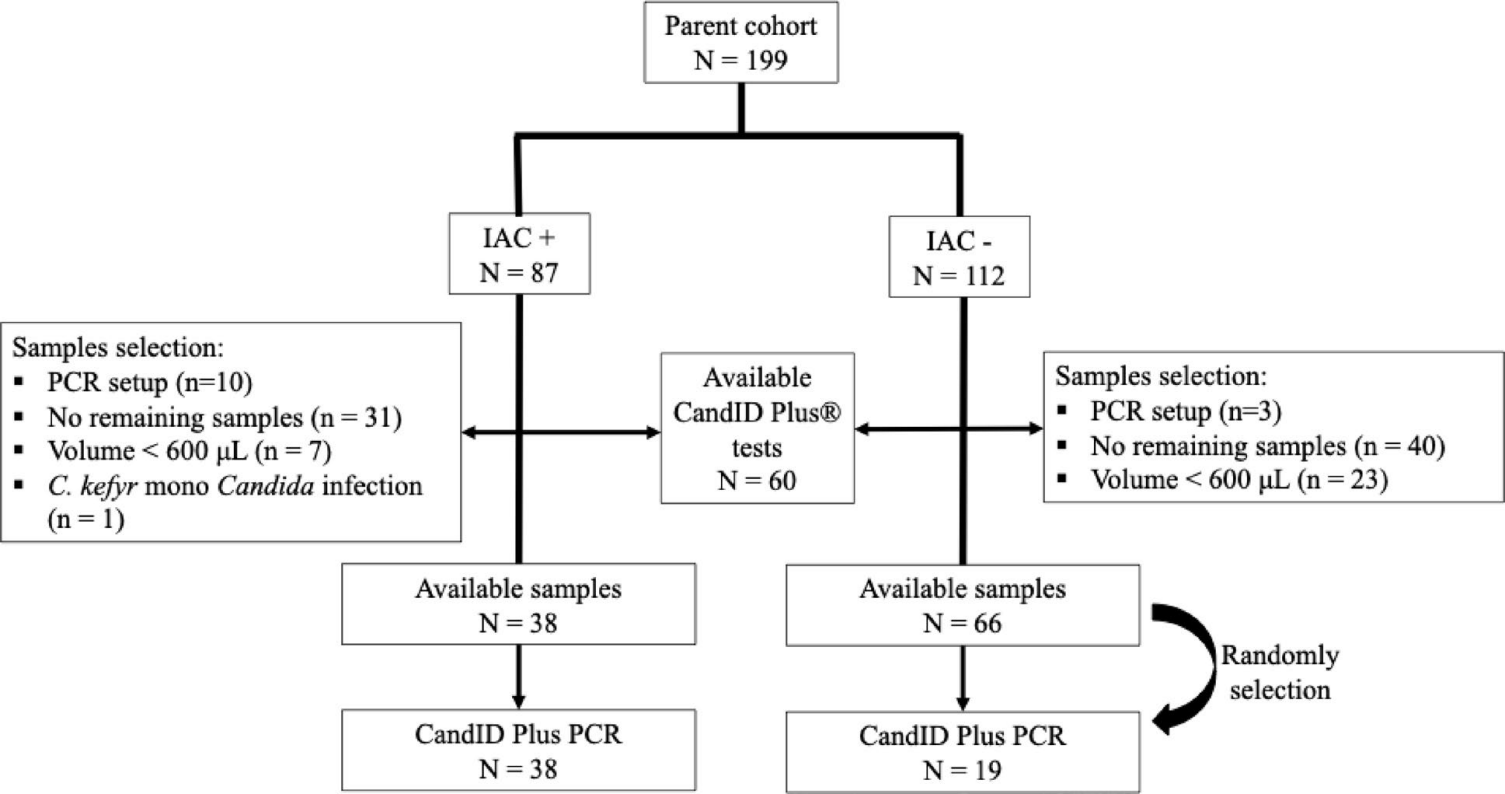
Flow chart of the study

Constraints related to storage, previous analyses, freeze–thaw cycles, residual volume, and PCR optimization reduced the eligible sample set to 38 culture-positive and 19 culture-negative specimens (see Flow Chart, Fig. 1).

The clinical characteristics of the 57 patients included in the analysis are presented in Table 1. Patients were predominantly male (65%), with a median age of 67 [55;74] years. Intra-abdominal infections were postoperative peritonitis in 49% of the patients (*n* = 28). Polymicrobial infection was reported in 74% of the patients. Only one patient reported candidemia. The median ICU length of stay was 8 [5;12] days, and the ICU mortality rate was 17% (*n* = 10).

**Table 1:** Clinical characteristics of critically ill patients

Variable	IAC (*n* = 38)	No IAC (*n* = 19)	*P* value
Age (years)	67 [61–76]	64 [53–74]	0.23
Male sex	24 (63)	13 (68)	0.69
Body mass index (kg/m^2^)	26.0 [23.2–29.4]	24.6 [19.4–26.7]	0.11
Knauss score			0.46
A B C	7 (18) 20 (53) 11 (29)	1 (5) 11 (58) 7 (37)	
McCabe score			0.16
1 2 3	20 (53) 14 (37) 4 (10)	6 (32) 12 (63) 1 (5)	
Comorbidities:			
Cardiovascular COPD Chronic renal insufficiency^1^ Cirrhosis	5 (13) 6 (16) 9 (24) 3 (8)	5 (26) 4 (21) 4 (21) 1(5)	0.27 0.72 1.0 1.0
Malnutrition^2^	28 (74)	17 (89)	0.30
Immunocompromised^3^	17 (45)	13 (68)	0.09
**ICU data**			
Admission SAPS II score	53 [46–65]	44 [38–58]	**0.04**
IAC diagnosis SOFA score	7 [4–9]	6 [4–10]	0.76
Septic shock (Sepsis 3)	26 (68)	10 (52)	0.24
Norepinephrine infusion	33 (87)	15 (79)	0.46
Invasive mechanical ventilation > 48 h	20 (53)	7 (37)	0.26
Renal replacement therapy	11 (39)	3 (27)	0.1
ICU mortality	8 (21)	2 (10)	0.46
**Peritonitis data**			
Postoperative peritonitis	17 (45)	11 (58)	0.35
Site of origin supra mesocolic	16 (42)	7 (37)	0.70
Mechanism			0.54
Perforation Necrosis Anastomosis leakage	19 (50) 6 (16) 13 (34)	12 (63) 3 (16) 4 (21)	
Presence of bacteria	26 (68)	16 (82)	0.20

The results expressed as the n (%) and median [IQR]

COPD: chronic obstructive pulmonary disease; IAC: intra-abdominal candidiasis; ICU: intensive care unit; RRT: renal replacement therapy; SAPS II: Simplified Acute Physiological Score; SOFA: Sequential Organ Failure Assessment score

^1^Chronic renal insufficiency: defined as an estimated glomerular filtration rate < 60 mL/min/1.73 m^2^

^2^Malnutrition: based on phenotype and etiology criteria from the ESPEN GLIM recommendations

^3^Immunocompromised: active cancer (solid tumor or haematological malignancy), organ transplant or bone marrow transplant, systemic and/or immune disease requiring immunosuppressive therapy, and receiving one or more immunosuppressive therapies for more than three months

In patients whose fungal cultures were positive, *C. albicans* was the most common *Candida* species (79%), followed by *C. glabrata (Nakaseomyces glabratus)* (10%) and *C. tropicalis* (5%). Six patients had positive cultures with two or more different *Candida* species. Only two out of 57 patients (3.5%) had received antifungal therapy before sample collection, suggesting a limited potential impact on culture yield in the present analysis. The time to positivity of the fungal culture was 2.5 days (min 0, max 5 days).

Table 2 presents the CT results of the OLM CandID Real-Time PCR for the whole cohort.

**Table 2:** Ct results in case of positive OLM candid Real-Time PCR

		IAC (*n* = 38)	No IAC (*n* = 19)
Patients with a PCR+		38	7
*C. albicans* PCR	n	32	3
	Ct	28.5 [23.3–31.5]	37.9[31.9–38.1]
*N. glabratus*^1^ PCR	n	9	0
	Ct	23.7 [20.5–33.0]	NA
*C. parapsilosis* PCR	n	3	1
	Ct	30.8 [24.6–35.7]	38.5
*C. tropicalis* PCR	n	10	2
	Ct	32.5 [24.4–34.6]	37.1[36.4–37.8]
*P. kudriavzevii*^2^ PCR	n	3	1
	Ct	31.1 [25.1–34.4]	29
*C. dubliniensis* PCR	n	2	0
	Ct	22.5 [18.5–26.6]	NA

Ct: cycle threshold; IAC: intra-abdominal candidiasis

The results are expressed as numbers and medians [IQRs]

^1^ *C. glabrata*

^2^ *C. krusei*

Receiver operating characteristic curve of OLM CandID Real-Time PCR with an optimal threshold to discriminate culture-positive cases from culture-negative/PCR-positive cases (using the Youden index) for *C. albicans* and *C. tropicalis* in critically ill patients with intra-abdominal candidiasis

Among culture-positive samples, PCR detected *Candida* DNA in all cases, resulting in 100% sensitivity for the qualitative detection of *Candida* spp. At the species level, full concordance was observed in all cases of monomicrobial *Candida* infection, with PCR identifying the same species as culture. In contrast, discrepancies were observed in polymicrobial infections and in some monomicrobial culture-positive cases. In six samples with polymicrobial fungal cultures, all cultured species were detected by PCR, but additional *Candida* species were also identified by PCR. In 11 samples with a single *Candida* species detected by culture, PCR identified the cultured species along with one or more additional species. Overall, species-level over-detection by PCR occurred in 13 culture-positive samples (34%). Detailed species-level comparisons are provided in Supplementary Table S1.

The peritoneal BDG concentration was above 45 pg/mL in all patients with positive fungal cultures and in 18 patients with negative fungal cultures (Supplementary Table S1).

The sBDG and pBDG results for all included samples are provided in the Supplementary Materials (Table S1). Contingency tables for fungal culture versus OLM CandID Real-Time PCR and fungal culture versus pBDG are provided in the Supplementary Materials (Tables S2 and S3).

### Primary objective

Using a binary interpretation, the OLM CandID Real-Time PCR demonstrated a sensitivity of 100% (95% CI 100–100) and a specificity of 60% (95% CI 38–81). The PPV and NPV were 82% (95% CI 71–93) and 100% (95% CI 100–100), respectively. PCR showed complete concordance with culture among positive samples, correctly identifying all 38 culture-positive cases. When culture-negative samples were included, the overall agreement between both methods reached 87.7%, yielding a Cohen’s kappa of 0.70, consistent with substantial agreement.

### Secondary objective

**Fig. 2:**
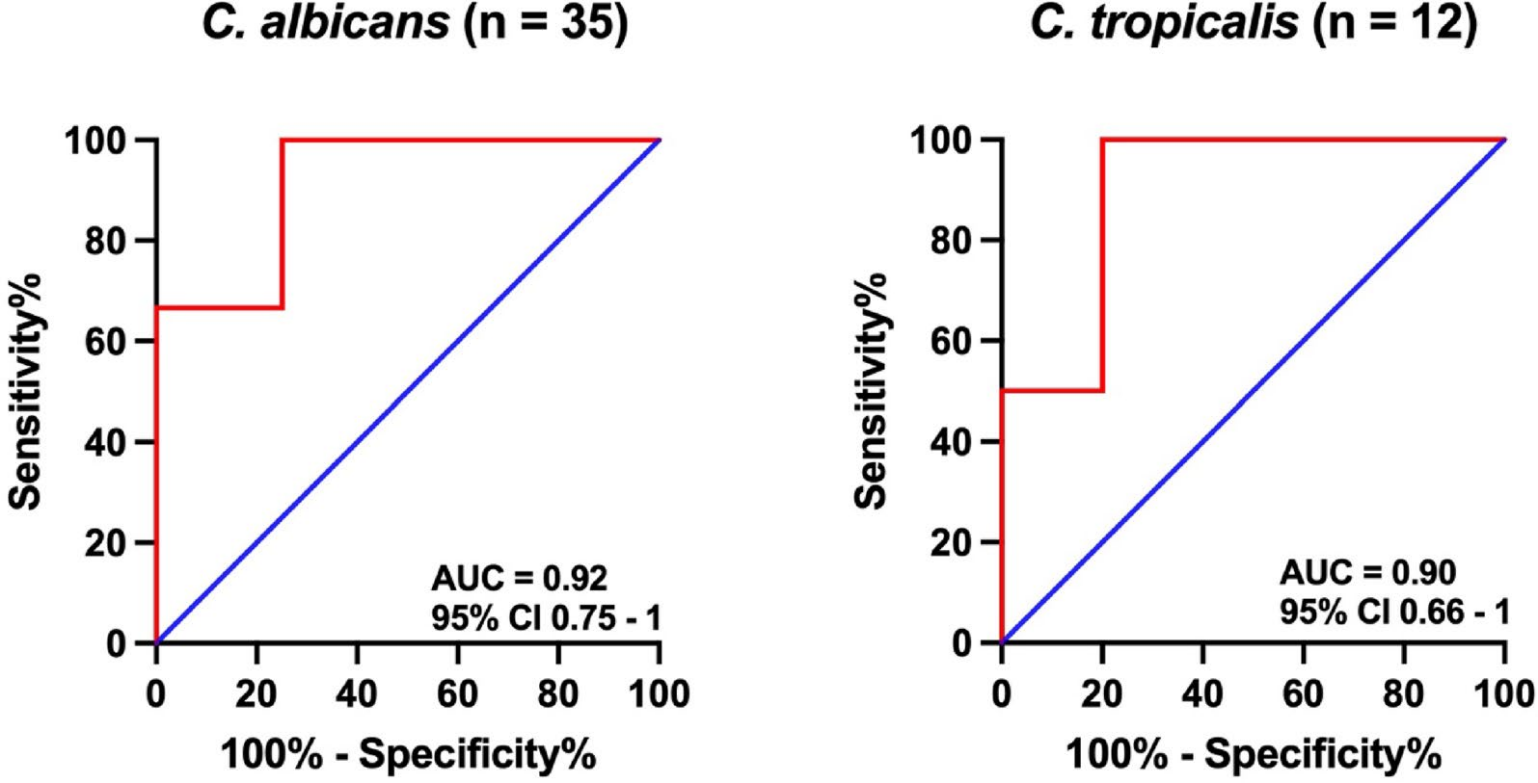
OLM CandID Real-Time PCR to detect *C. albicans* and *C. tropicalis* in the peritoneal fluid of critically ill patients with suspicion of intra-abdominal candidiasis

ROC-based Ct determination and subsequent reclassification were evaluated for *C. albicans* (*n* = 35) and *tropicalis* (*n* = 12) (Fig. 2).

For *C. albicans*, the ROC AUC was 0.92 [0.75–1]. When a Ct cutoff value of 30.75 (determined by the highest Youden index) was used, the specificity and PPV were 100% (95% CI 100–100). The sensitivity and NPV were 69% (95% CI 53–85) and 23% (95% CI 0–46), respectively.

For *C. tropicalis*, the ROC AUC was 0.90 [0.66–1]. When a Ct cutoff value of 34.63 was used, the specificity and PPV were 100% (95% CI 100–100). The sensitivity and NPV were 70% (95% CI 42–98) and 40% (95% CI 0–83), respectively.

The diagnostic performance of pBDG using a binary approach demonstrated a sensitivity of 100% (95% CI 100–100) and a specificity of 10% (95% CI 0–23). The PPV and NPV were 67% (95% CI 54–80) and 100% (95% CI 100–100), respectively. The ROC AUC was 0.54. Given that the AUC did not exceed 0.80, no attempt was made to determine an optimal cutoff, since such analysis would likely yield nonmeaningful results.

## Discussion

This multicenter study is the first to evaluate the diagnostic performance of the CandID Real-Time PCR (OLM Diagnostics Ltd., Braintree, UK) assay applied directly to peritoneal fluid in critically ill patients with suspected or confirmed intra-abdominal candidiasis. Within the panel of targeted species, the assay showed high analytical performance, supporting its potential value for rapid species-level characterization within its detection spectrum. *Candida* DNA was detected by PCR in all culture-positive samples, with full species-level concordance in monomicrobial infections. In contrast, in polymicrobial infections and in a subset of monomicrobial cases, PCR identified additional *Candida* species beyond those recovered by culture, resulting in species-level over-detection in approximately one-third of culture-positive samples. A second key observation is that PCR-positive/culture-negative results were predominantly associated with Ct values above 30, a range typically linked to very low fungal loads [27]. Whether these findings reflect true infections detected by a more sensitive technique or low-level signals without pathogenic significance remains uncertain. This ambiguity underscores the need for complementary biomarkers to refine interpretation, particularly because the pathogenic role of *Candida* in peritoneal fluid itself remains debated [28, 29].

Previous investigations using molecular approaches in the peritoneal fluid for IAC diagnosis reported encouraging results but with noteworthy methodological differences [21, 23]. Xie et al. [23] used broad ITS-PCR with sequencing and reported moderate sensitivity (64.7%) and high specificity (89.4%), which is consistent with the accuracy observed in our cohort. However, their single-center design, requirement for sequencing, and lack of quantitative assessment limit the suitability of their approach for rapid diagnostics in the ICU. Similarly, Corrales et al. [21] reported good concordance between a PCR microarray and culture, reinforcing the potential value of molecular tools. Although promising for bloodstream infections, the T2Candida Panel has shown limited diagnostic performance for IAC and is not validated for peritoneal fluid, with restricted clinical availability further limiting its use [24, 30, 31]. Our work adds multicenter validation, uses a ready-to-use assay with species-level identification, and introduces quantitative interpretation. The Ct thresholds identified here (30.75 for *C. albicans* and 34.63 for *C. tropicalis*) align closely with previously published real-time PCR data [27] but also highlight a fundamental diagnostic dilemma: distinguishing improved sensitivity from overdetection of low fungal burdens suggestive of colonization or nonspecific amplification.

In this context, ancillary biomarkers were examined as potential contextual tools for interpreting PCR-positive/culture-negative results. In the present study, peritoneal β-D-glucan (pBDG) was the only available complementary marker. Because these samples originated from the same cohort in which the 45 pg/mL pBDG cutoff for distinguishing IAC from non-IAC had been originally derived, all culture-positive cases were expected to exceed this threshold, and overlap of pBDG values among culture-negative patients was already anticipated. Consequently, pBDG did not allow reliable discrimination of discordant cases, illustrating how biomarker interpretation is highly dependent on the population in which diagnostic thresholds are generated.

Serum and peritoneal BDG also lack specificity in critically ill patients due to frequent false positives associated with invasive procedures, broad-spectrum antibiotics, and polymicrobial infections [7, 12]. Furthermore, daily sBDG testing is not consistently available across centers and often requires more than 48 h [32], whereas pBDG remains a research-use test with technical limitations affecting inter-study comparability [12, 33]. Although such biomarkers may contribute to disease exclusion in selected settings, they do not provide species-level identification, which is increasingly recognized as critical for early, targeted antifungal decision-making.

Despite growing interest in molecular diagnostics, no PCR assay is currently recommended in international guidelines to optimize IAC detection [17]. Rapid, accurate, species-specific PCR directly performed on peritoneal fluid therefore represents a pragmatic strategy aligned with research priorities for early antifungal management [16]. Although combining *Candida* PCR with sBDG has been proposed in high-risk candidemia [15], this strategy could not be formally assessed in the present post hoc analysis. Consistently, among culture-negative/PCR-positive samples, pBDG concentrations exceeded 45 pg/mL in 6 of 7 cases (86%), and sBDG concentrations were above the positivity threshold in 3 of 7 cases (42%), further highlighting the limited discriminatory value of BDG in this setting.

Although the present post hoc analysis did not directly evaluate time-to-result, the OLM CandID Real-Time PCR assay is capable of delivering results within 2–3 h once a sample is loaded onto the InGenius platform. In real-world clinical scenarios, considering sample transport and workflow logistics, results could reasonably be available within 6–8 h, and in any case within 24 h. Rapid availability of species-level results could potentially allow earlier targeted antifungal therapy, limit unnecessary exposure to empiric antifungal agents, and reduce associated drug costs, although these outcomes remain to be formally assessed in prospective studies.

This study has several strengths. It is the largest multicenter evaluation of a targeted species-level molecular assay applied directly to peritoneal fluid. The assay covers the majority of *Candida* spp. implicated in IAC and was technically reliable, with few analytical failures. It is also the first to propose Ct thresholds that provide initial insights into potentially discriminating between clinically meaningful and detection of low-level fungal DNA without established pathogenic significance, addressing one of the principal limitations of fungal PCR. However, these secondary ROC analyses based on Ct values should be viewed as exploratory: they were restricted to a limited number of *Candida* species and were not designed to assess associations with clinical outcome surrogates, which constrains their incremental interpretative value.

Limitations must be acknowledged. First, the limited number of culture-negative samples restricts the precision of specificity estimates and constraints the interpretation of PCR-positive/culture-negative results. In addition, the post hoc design, small species-specific sample sizes, and absence of external validation mean that the proposed Ct thresholds are intended for hypothesis generation rather than clinical decision-making. Preexisting sample constraints limited species-specific quantitative analyses, and the low number of isolates prevented robust ROC modeling across multiple *Candida* species. Because the sample set was enriched for culture-positive specimens, predictive values (PPV and NPV) should be interpreted within this context and may not reflect those in a general ICU population. From an operational perspective, the post hoc design also precluded assessment of the real-time applicability of the assay within routine laboratory workflows. In terms of analytical scope, the PCR detection panel does not include *C. kefyr (Kluyveromyces marxianus)*, although this species accounts for less than 5% of the reported isolates in major IAC cohorts [3], and all patients undergo fungal culture, minimizing the risk of missed diagnoses. Finally, although samples were stored at − 20 °C during the analytical phase, this condition reflects routine laboratory practice for DNA-based assays and is unlikely to have significantly impacted PCR performance.

In terms of generalizability, recent expert recommendations advocate early integration of molecular tools into IAC diagnostic strategies [16]. The OLM CandID Real-Time PCR assay is particularly suited for this purpose because it amplifies directly from peritoneal fluid, bypassing the need for media pre-enrichment steps required by other molecular platforms. While the assay targets a defined panel of clinically relevant *Candida* species rather than providing universal fungal or genus-level *Candida* detection, this spectrum encompasses most species reported in IAC cohorts and allows rapid, species-specific information that cannot be obtained with antigen-based tests.

Given its a priori strong analytical performance and potential to support antifungal stewardship, this assay could be implemented in centers equipped with standard molecular facilities. However, the present findings are exploratory, and prospective validation is required to confirm the clinical utility of the assay and to guide interpretation of PCR-positive/culture-negative results. While this study does not evaluate antifungal decision-making, patient-centered outcomes, or stewardship impact, it provides foundational data that can inform the design of future prospective studies.

## Conclusion

In this multicenter post hoc analysis, species-directed CandID Real-Time PCR (OLM Diagnostics, Braintree, UK) applied directly to peritoneal fluid demonstrated strong analytical performance for the detection of *Candida* spp. in critically ill patients with suspected intra-abdominal candidiasis. High concordance was observed for both culture-positive/PCR-positive and culture-negative/PCR-negative cases, supporting the potential utility of this approach for excluding or confirming the presence of *Candida* in peritoneal samples.

Discordant PCR-positive/culture-negative results, often associated with high Ct values, may reflect undetected infection, detection of low-level fungal DNA without established pathogenic significance, or analytical over-detection. While complementary biomarkers may contribute to result interpretation, the clinical relevance of Ct-based stratification remains exploratory. Prospective studies are therefore required to validate Ct thresholds, assess real-time implementation, and determine the impact of PCR-guided strategies on antifungal management and patient-centered outcomes.

Overall, these findings support further evaluation of rapid, quantitative, species-targeted PCR applied to peritoneal fluid as part of integrated diagnostic strategies for intra-abdominal candidiasis.

## Take-home messages

- The OLM CandID Real-Time PCR assay can be directly applied to peritoneal fluid with excellent technical feasibility, enabling rapid, species-level *Candida* detection without pre-enrichment steps.
- In this post hoc, proof-of-concept analysis, PCR showed high concordance with fungal culture within its target spectrum, suggesting potential utility for rapidly confirming or excluding the presence of *Candida* spp. in peritoneal fluid, while acknowledging that clinical impact was not assessed.
- PCR-positive/culture-negative results occurred predominantly at high Ct values, suggesting low fungal burden or analytical over-detection; these observations are exploratory and underscore the need for complementary biomarkers and prospective clinical validation.

## Supplementary Information

Supplementary Material 1.

## Data Availability

The dataset used and/or analyzed during the current study is available from the corresponding author upon reasonable request.

## Abbreviations

AUC: Area under the curve
Ct: Cycle threshold
IAC: Intra-abdominal candidiasis
ICU: Intensive care unit
pBDG: Peritoneal 1.3 beta-d-glucan
PCR: Polymerase chain reaction
ROC: Receiver operating characteristic
sBDG: Serum 1.3 beta-d-glucan
