# Supplementary material for: Analytical performance of species-targeted quantitative PCR for intra-abdominal candidiasis in critically ill patients: a proof-of-concept post hoc analysis from the pBDG2 multicenter study
Source: Crit Care. 2026 Feb 10;30:117. doi: 10.1186/s13054-026-05882-5 (PMC12990629; doi:10.1186/s13054-026-05882-5)
Supplement: Supplementary file 1 — Supplementary Material 1. [file 13054_2026_5882_MOESM1_ESM.docx]

**Supplementary materials**

**Supplementary Table S1:** Individual sample-level microbiological and biomarker data

| Patients | Fungal culture results | PCR results | Ct | sBDG D1  pg/mL | sBDG D3  pg/mL | pBDG  pg/mL |
| --- | --- | --- | --- | --- | --- | --- |
| 1 | *C. albicans* | *C. albicans*  *P. kudriavzevii* | 23.01  34.4 | 130 | 49 | 4045 |
| 2 | *P. kudriavzevii*  *N. glabratus*  *C. albicans* | *P. kudriavzevii*  *N. glabratus*  *C. albicans*  *C. tropicalis* | 27.04  26.95  24.43  31.11 | 434 | 298 | 2218 |
| 3 | *C. albicans* | *C. albicans* | 33.06 | - | - | 2019 |
| 4 | *C. tropicalis* | *C. tropicalis* | 22.59 | - | - | 623.2 |
| 5 | *C. albicans* | *C. albicans* | 28.74 | 110 | 44 | 4885 |
| 6 | *C. albicans* | *C. albicans* | 30.75 | 37 | 57 | 1350 |
| 7 | *C. albicans*  *N. glabratus* | *C. albicans*  *N. glabratus* | 30.75  20.55 | 45 | 18 | 2674 |
| 8 | *C. albicans*  *N. glabratus* | *C. albicans*  *N. glabratus*  *C. tropicalis* | 29.04  33.05  27.55 | 354 | 520 | 13540 |
| 9 | *C. albicans* | *C. albicans* | 20.31 | 517 | 488 | 647.8 |
| 10 | *C. albicans* | *C. albicans*  *C. tropicalis* | 30.14  37.55 | 7 | 31 | 2950 |
| 11 | *C. albicans* | *C. albicans* | 24.34 | 90 | 220 | 283.8 |
| 12 | *N. glabratus* | *N. glabratus*  *C. albicans* | 23.69  36.2 | 154 | 129 | 54.06 |
| 13 | *C. albicans* | *C. albicans* | 21.54 | 149 | 183 | 426.1 |
| 14 | *C. albicans* | *C. albicans* | 24.9 | 80 | 49 | 691.5 |
| 15 | *C. albicans* | *C. albicans* | 34.83 | 38 | 13 | 186.6 |
| 16 | *C. albicans* | *C. albicans* | 28.82 | 379 | 303 | 1350 |
| 17 | *C. albicans* | *C. albicans* | 21.1 | 809 | 1500 | 2674 |
| 18 | *N. glabratus* | *N. glabratus*  *C. albicans* | 34.85  36.77 | - | - | 82.43 |
| 19 | *C. albicans*  *K. marxianus* | *C. albicans*  **NA** | 23.71 | 5000 | 1038 | 1012 |
| 20 | *C. albicans*  *N. glabratus* | *C. albicans*  *N. glabratus* | 22.79  21.46 | 44 | 4 | 1578 |
| 21 | *C. tropicalis* | *C. tropicalis*  *C. parapsilosis* | 19.59  30.85 | 211 | 148 | 283.8 |
| 22 | *C. albicans* | *C. albicans* | 19.48 | 4848 | 3022 | 2432 |
| 23 | *C. albicans* | *C. albicans* | 33.41 | 122 | 202 | 369.3 |
| 24 | *C. parapsilosis* | *C. parapsilosis* | 24.62 | 38 | 15 | 91.11 |
| 25 | *N. glabratus*  *K. marxianus* | *N. glabratus*  **NA** | 14.25  **NA** | 136 | 130 | 522.5 |
| 26 | *C. albicans* | *C. albicans*  *C. parapsilosis*  *C. tropicalis*  *P. kudriavzevii* | 22.01  35.72  34.63  25.13 | 90 | - | 157.6 |
| 27 | *C. albicans* | *C. albicans*  *C. tropicalis* | 29.19  37.28 | **3** | **2.5** | 1350 |
| 28 | *C. albicans* | *C. albicans* | 28.17 | **6** | **8** | 61.23 |
| 29 | *C. albicans* | *C. albicans*  *C. tropicalis* | 28.34  32.51 | **13** | **7** | 101.2 |
| 30 | *C. albicans* | *C. albicans* | 35.35 | **22** | **18** | 61.23 |
| 31 | *C. albicans* | *C. albicans*  *C. tropicalis* | 29.37  32.79 | **3** | **3** | 5095 |
| 32 | *C. albicans* | *C. albicans* | 26.01 | **18** | **15** | 19090 |
| 33 | *C. albicans* | *C. albicans* | 21.07 | **9** | **10** | 2218 |
| 34* | *C. albicans* | *C. albicans* | 18.01 | **3** | **3** | 886.1 |
| 35 | *C. albicans* | *C. albicans* | 24.72 | **3** | **3** | 1165 |
| 36 | *C. albicans* | *C. albicans*  *N. glabratus* | 32.16  37.07 | 30 | 30 | 360 |
| 37 | *N. glabratus* | *N. glabratus*  *C. tropicalis*  *C. dubliensis* | 17.14  32.45  26.56 | - | 146 | 180.2 |
| 38* | *C. albicans* | *C. albicans* | 37.11 | 86.9 | - | 119.6 |
|  |  |  |  |  |  |  |
| 1 | Negative | *C. tropicalis* | 38.49 | - | - | 11.16 |
| 2 | Negative | *P. kudriavzevii* | 29.04 | 10 | 47 | 57.48 |
| 3 | Negative | Negative | **NA** | 61 | 25 | 7467 |
| 4 | Negative | *C. albicans* | 37.87 | 125 | 107 | 4045 |
| 5 | Negative | Negative | **NA** | - | - | 405.9 |
| 6 | Negative | Negative | **NA** | - | - | 283.8 |
| 7 | Negative | Negative | **NA** | 508 | 431 | 522.5 |
| 8 | Negative | *C. tropicalis* | 36.44 | 100 | 44 | 387 |
| 9 | Negative | Negative | **NA** | - | 26 | 62.56 |
| 10 | Negative | Negative | **NA** | 141 | 136 | 522.5 |
| 11 | Negative | *C. albicans* | 38.09 | 48 | 60 | 2674 |
| 12 | Negative | Negative | **NA** | - | - | 162.8 |
| 13 | Negative | Negative | **NA** | **5** | **3** | 1165 |
| 14 | Negative | Negative | **NA** | **3** | **3** | 6539 |
| 15 | Negative | *C. tropicalis* | 37.82 | **1** | **1** | 2432 |
| 16 | Negative | Negative | **NA** | **3** | **3** | 91.11 |
| 17 | Negative | Negative | **NA** | **3** | **3** | 54.06 |
| 18 | Negative | Negative | **NA** | **3** | **3** | 3.24 |
| 19 | Negative | *C. albicans* | 31.93 | **23** | **38** | 8577 |

**Legend:** *N. glabratus (Nakaseomyces), P. kudriavzevii (Pichia) and* *K. marxianus (Kluyveromyces) refer to C. glabrata, C. krusei and C. Kefyr, respectively, according to the new nomenclature.*

**Abbreviations:** - : missing data; *: prior antifungal exposure; Ct: cycle threshold; D1 – D3: Day 1, 24h after the surgery / Day 3: 72h after the surgery; NA : not applicable; pBDG: Peritoneal 1.3-beta-d-glucan measured using the beta-d-glucan test (Fujifilm Wako Chemicals Europe, Neuss, Germany); sBDG: serum 1.3-beta-d-glucan measured using the beta-d-glucan test (Fujifilm Wako Chemicals Europe, Neuss, Germany); in **bold** or Fungitell® beta-D-glucan (Associated of Cape Cod, East Falmouth, Inc., United States of America).

**Supplementary Table S2:** Contingency table (*Candida* culture/PCR*).

|  | *Candida* culture | |  |
| --- | --- | --- | --- |
| PCR | Negative | Positive | Total |
| Negative | 12 | **0** | 12 |
| Positive | 7 | **38** | 45 |
| Total | 19 | **38** | 57 |

**Abbreviations:** PCR: polymerase chain reaction.

*OLM CandID Real-Time PCR (OLM Diagnostics, Braintree, UK).

Binary diagnostic performance: PCR results were classified as positive or negative based solely on the presence or absence of an amplification signal, without applying any Ct threshold

**Supplementary Table S3:** Contingency table (*Candida* culture/peritoneal 1.3-beta-d-glucan)

|  | *Candida* culture | |  |
| --- | --- | --- | --- |
| pBDG | Negative | Positive | Total |
| ≤ 45 pg/ml | 2 | 0 | 2 |
| > 45 pg/ml | 17 | 38 | 55 |
| Total | 19 | 38 | 57 |

**Abbreviation**: pBDG: Peritoneal 1.3-beta-d-glucan measured using the beta-d-glucan test (Fujifilm Wako Chemicals Europe, Neuss, Germany).
